# The complete mitochondrial genome of *Lunella coronata correensis*

**DOI:** 10.1080/23802359.2019.1704183

**Published:** 2020-01-08

**Authors:** Jiacheng Yang, Jianye Zhu, Ling Shi, Zhuo Li, Dandan Zhang, Ying Tian, Zhenlin Hao

**Affiliations:** a Key Laboratory of Mariculture & Stock Enhancement in North China’s Sea, Ministry of Agriculture and Rural Affairs, Dalian Ocean University, Dalian, PR China;; b Dalian Shell Museum, Dalian, PR China

**Keywords:** Mitochondrial genome, Turbinidae, *Lunella coronata correensis*

## Abstract

The complete mitochondrial (mt) genome of *Lunella coronata correensis* was determined using genome walking techniques in this study. The total length of the mt genome sequence of *L. coronata correensis* was 16,551 bp, including 13 protein-coding genes, 23 transfer RNA genes, and 2 ribosomal RNA genes. The overall composition of the mitogenome was estimated to be 33.1% for A, 35.3% for T, 13.7% for C, and 19.9% for G, respectively, indicating that an A + T (68.4%)-rich feature occurs in the *L. coronata correensis* mitogenome. The phylogenetic relationships of 12 mollusk species were constructed based on the complete mtDNA sequences by the neighbor-joining method using MEGA version 7.0 and DNAMAN version 6.0 software.

The *Lunella coronata correensis* belongs to the family Turbinidae, and could be found in northern parts Yellow Sea and Bohai Sea of China. The population of wild *L. coronata correensis* is very small, and the research about *L. coronata correensis* is rare. In order to provide some biological data for *L. coronata correensis,* it is necessary to carry out some wild resource investigation and germplasm analysis. In this study, we report the complete sequence of mitochondrial (mt) genome for *L. coronata correensis* (GenBank accession no. MN604179). The findings will provide useful information for further studies on population genetics, phylogenetic construction and other relevant studies in *L. coronata correensis*.

One *L. coronata correensis* individual was collected from Dalian Sea area, Liaoning Province, China (39°11′23″N, 124°47′24″E). The total genomic DNA was extracted from foot muscle by a modification of standard phenol-chloroform procedure. The complete mitogenome of *L. coronata correensis* was sequenced by primer walking. The gene annotation was performed following the methods described by Hao et al. (2016) and Online software (GeneMarker, tRNAscan, ORFfinder). At present, The *L. coronata correensis* specimens were stored in the Key Laboratory of Mariculture & Stock Enhancement in North China’s Sea, Ministry of Agriculture and Rural Affairs, PR China, Dalian Ocean University, Dalian, 116023, Liaoning, China. The specimens were numbered DLOU -KLM-SSH06 to DLOU-KLM-SSH09.

The total length of the hybrid of *L. coronata correensis* mt genome was 16,551 bp, with the base composition of 33.1% A, 35.3% T, 13.7% C, and 19.9% G. It comprised 2 ribosomal RNA genes, 13 protein-coding genes, and 23 transfer RNA genes. All the mitogenome genes were encoded on the light strand except for 11 tRNA genes (*tRNA-Asp*, *tRNA-Glu*, *tRNA-Gly*, *tRNA-Ala*, *tRNA-Arg*, *tRNA-Asn*, *tRNA-Ser*, *tRNA-Ile*, *tRNA-(Lys/Asn)*, and *tRNA-Thr*).

The phylogenetic analysis showed that the complete mt sequence of *L. coronata correensis* was phylogenetically closer to *Bolma rugosa by* 71.76% bootstrap support, and formed the Turbinidae clade (Figure 1). Our result was consistent with the previous researches, both in traditional morphological and molecular-based phylogeny studies. We expect that the present result can contribute to construct molecular identification of this species and be helpful to explore the phylogeny of Muricidae.

**Figure 1.**
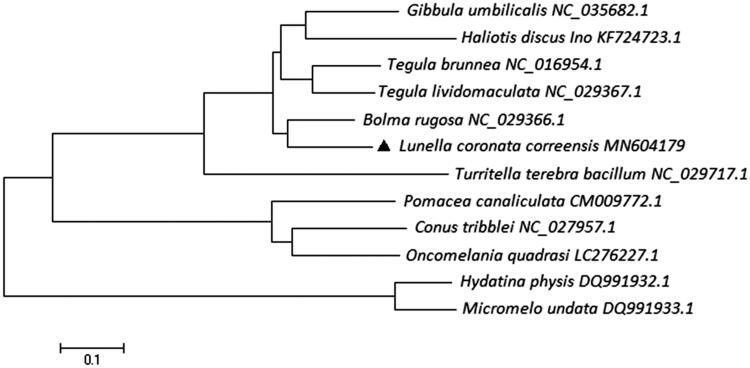
Consensus neighbor-joining tree based on the complete mitochondrial sequence of *L. coronata correensis* and other 11 mollusk species. The phylogenetic tree was constructed using MEGA 7.0 and DNAMAN 6.0 software by the neighbor-joining method. The numbers at the tree nodes indicate the percentage of bootstrapping after 1000 replicates.
